# Accelerated Synthesis of Borophane (HB) Sheets through HCl-Assisted Ion-Exchange Reaction with YCrB_4_

**DOI:** 10.3390/molecules28072985

**Published:** 2023-03-27

**Authors:** Xiaoni Zhang, Miwa Hikichi, Takushi Iimori, Yuki Tsujikawa, Mei Yuan, Masafumi Horio, Kunio Yubuta, Fumio Komori, Masahiro Miyauchi, Takahiro Kondo, Iwao Matsuda

**Affiliations:** 1 Institute for Solid State Physics (ISSP), The University of Tokyo, Kashiwa 277-8581, Japan; 2 Faculty of Pure and Applied Sciences, University of Tsukuba, Tsukuba 305-8571, Japan; 3 Faculty of Engineering, Kyushu University, Fukuoka 819-0395, Japan; 4 Department of Materials Science and Engineering, School of Materials and Chemical Technology, Tokyo Institute of Technology, 2-12-1 Ookayama, Meguro-ku, Tokyo 152-8552, Japan

**Keywords:** two-dimensional material, hydrogen boride (HB), borophane, ion exchange, infrared spectroscopy

## Abstract

We present an enhanced method for synthesizing sheets of borophane. Despite the challenges associated with low efficiency, we discovered that incorporating hydrochloric acid into the ion-exchange reaction significantly improved the production yield from 20% to over 50%. After a thorough examination of the reaction, we gained insight into the underlying mechanisms and found that the use of hydrochloric acid provides two key benefits: accelerated production of borophene and isolation of high-purity products. This method has the potential to pave the way for the production of novel topological 2D materials with potential industrial applications.

## 1. Introduction

Explorations of two-dimensional (2D) materials beyond graphene have pushed the cutting edge of materials science, leading to the development of next-generation devices [1,2,3,4,5,6]. Recently, atomic sheets of boron, known as borophene, were synthesized as a promising counterpart to graphene [7,8,9]. In contrast to carbon, boron atoms can combine under diverse bonding schemes, resulting in rich allotropes that display a variety of electronic structures [10,11]. The unique characteristics of 2D boron offer exotic quantum states and intriguing features, such as anisotropic metallicity and phonon-mediated superconductivity [12,13,14]. However, the layers of borophene require epitaxial growth on a crystal surface under a ultrahigh vacuum, a process that is prone to oxidization under ambient conditions [11,12,13,14,15]. This disadvantage makes novel materials made from borophene difficult for industrial applications.

To passivate the boron atoms chemically and to extract the free-standing layer, the synthesis of hydrogen boride (HB, borophane, or hydrogenated borophene) has recently come under scrutiny through a variety of hydrogenation techniques. The most simple and energy-saving approach is the liquid exfoliation method at room temperature, which is associated with an ion-exchange reaction of materials that incorporate a 2D framework of boron [16,17,18,19,20]. In metal boride crystals, such as ${\mathrm{MgB}}_{2}$ and ${\mathrm{YCrB}}_{4}$, borophene layers are sandwiched by metal atoms. The ion-exchange reaction proceeds through the deintercalation of the metal cations and hydrogenation of the boron layers, resulting in the formation and subsequent extraction of HB layers. The hydrogen terminations are held on both sides of the free-standing layer and make the material robust within ambient environments. This extreme surface treatment of an atomic layer was achieved with ${\mathrm{MgB}}_{2}$ crystals in forming the honeycomb borophane composed of six-membered (hexagon) rings of boron [16,17]. Recently, a different borophane was synthesized by the same method from the ${\mathrm{YCrB}}_{4}$ crystals [18,20], which is a Dirac nodal-loop semimetal characterized by a $Z_{2}$ topology [20,21]. The “topological borophane” is made of five-membered (pentagon) and seven-membered (heptagon) rings, arranged to exhibit non-symmorphic symmetry. For simplicity, we abbreviate the name of the material as “5,7-HB”. The atomic structure of 5,7-HB, as verified by calculation, indicates that the material has a low area mass density of only 21 % compared to graphene, highlighting its potential as a versatile material for various applications [21]. With the existence of various 2D polymorphs of boron known to exist in crystals of metallic borides, numerous borophene structures are possible [10].

The basic procedure of the ion-exchange reaction is to mix the metal boride crystals and ion resins in an organic solvent such as acetonitrile [16,17,18,19,20]. The reaction typically results in a production yield of 42.3% and takes 2–4 days at room temperature under the atmospheric $N_{2}$ pressure, to obtain macroscopic samples of, for example, honeycomb HB developed from ${\mathrm{MgB}}_{2}$ [16]. This method was also found to be applicable to ${\mathrm{YCrB}}_{4}$ crystals [18,19,20]; the synthesis generally required over three weeks of reaction time to achieve a 20% production yield. The simple mixing process has the potential to shorten the reaction time and increase efficiency. In the research on honeycomb HB synthesis, a 50% production yield was achieved within two hours by adding formic acid to the solution [22]. This improvement is believed to occur because the acid plays the role of a mediator for efficient proton exchange between boride and resin.

**Figure 1:**
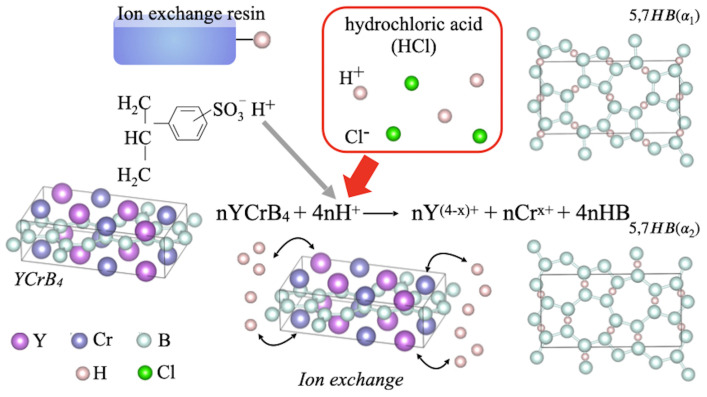
Concept of the synthesis of HB sheets with the 5- and 7-membered rings (5,7-HB) from a ${\mathrm{YCrB}}_{4}$ crystal through the addition of hydrochloric acid (HCl).

In the present research, we examined the acidic effects from ion-exchange reactions during the synthesis of 5,7-HB from a ${\mathrm{YCrB}}_{4}$ crystal, as schematically drawn in Figure 1. The reaction was accelerated by adding hydrochloric acid to obtain macroscopic samples and a yield of >50% after several hours, whereas, by comparison, the original ion-exchange reaction provided a production yield of 20% after three weeks. Quantitative characterizations were made through X-ray photoelectron spectroscopy (XPS) and infrared-ray absorption spectroscopy (IRAS). Microscopic observations were made to evaluate the size of the product sheet. Details of liquid exfoliation were uncovered at separate steps in the synthesis. When subjected to the ion-exchange reaction, the metal atoms Y and Cr from the mother material ${\mathrm{YCrB}}_{4}$ become metal chlorides and precipitate as residues with byproducts, namely boric acid and remnants of the mother material, ${\mathrm{YCrB}}_{4}$. The high-speed reaction and ease of isolating the pure product offer a new strategy for the efficient synthesis and mass production of functional borophene sheets.

## 2. Results and Discussion

### 2.1. Synthesis Process

A sheet of HB with a boron network of 5- and 7-membered rings can be prepared from crystals of ${\mathrm{YCrB}}_{4}$ through the liquid exfoliation method with an ion-exchange reaction [18,19,20]. The reaction is described as

*n*YCrB_4_ + 4*n*H^+^ → *n*Y^(4−x)+^ + *n*Cr^x+^ + 4*n*HB,

where HB represents a sheet of hydrogen boride or borophane. The reaction yield of the reaction can be evaluated by a ratio between *n* mol ${\mathrm{YCrB}}_{4}$ and 4*n* mol HB.

**Figure 2:**
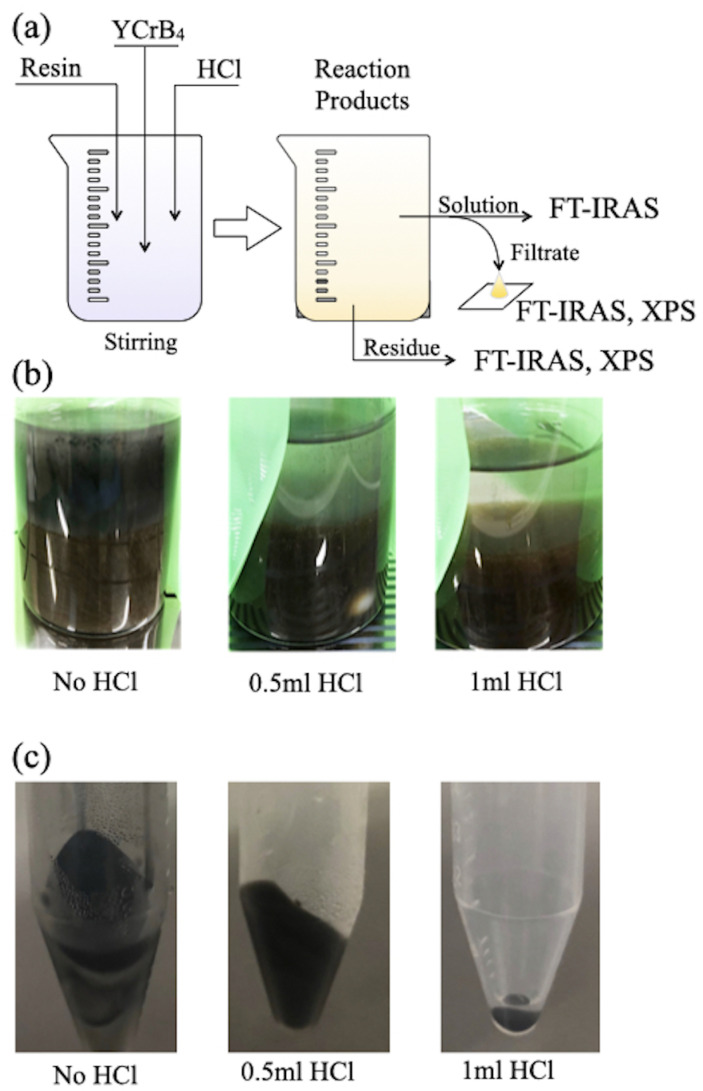
(**a**) Schematic drawing of the synthesis and characterization processes; (**b**,**c**) photos of (**b**) the reaction solutions and (**c**) the residue after three days of reaction with different amounts of hydrochloric acid.

Figure 2a illustrates the experimental procedures. The ion-exchange reaction was performed by stirring ${\mathrm{YCrB}}_{4}$ crystals and ion-exchange resin in a solvent containing hydrochloric acid. The resulting reaction products were filtered and separated into solution and residues. The solution was then filtered again to remove any boric acid and other possible by-products. Fourier-transform (FT)-IRAS measurements on the solution, residues, and filtrate, while XPS measurements were conducted on the filtrate and residues. During the ion-exchange reaction, 0.2 mL samples of the solutions were taken using a volumetric pipette after reaction times lasting one hour, three hours, one day, and three days to conduct FT-IRAS measurements. We note that the solution contained layers of 5,7-HB that could be extracted through centrifugation (5000 rmp for 5 min) and filtration. The filtrate was also dropped and dried on a sample holder in Ar atmosphere for the XPS analysis. Figure 2b shows a series of photos taken after three days of reaction. Transparent and dark regions are observed in the sample depending on the amount of hydrochloric acid used. After removal of the ion-exchange resin and centrifugation, the sample was separated into a residue/precipitate [Figure 2c] and a solution that eventually provided the borophene sheet after filtration.

**Figure 3:**
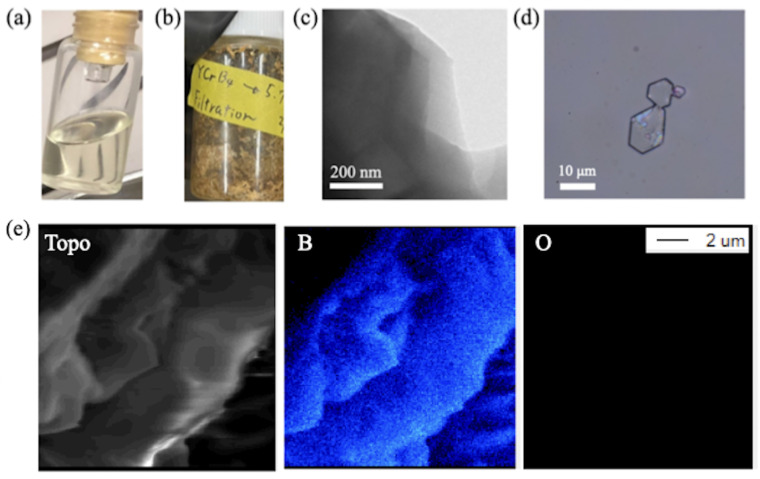
(**a**) A photo of the filtrate sample, synthesized by the addition of 1 mL HCl; (**b**) a photo of the powder sample after the drying treatment of (**a**); (**c**) an optical microscopy image of the single HB flake obtained after spin-coating of (**a**); (**d**) TEM images of the powder sample of (**b**). (**e**) SEM-EPMA images of the topographic (Topo) map and elemental maps of boron (B) and oxygen (O) of the HB flake.

Confirmations of the sheet morphology were made by two imaging approaches. For the first case, we isolated a single flake of the HB sheet from the sample filtrate (Figure 3a) by the spin-coating method, followed by observation with an optical microscope. As shown in Figure 3c, the sheet was clearly visible and had a size of 10 µm. For the second case, we dried the sample filtrate into powder (Figure 3b) and conducted the observation with a transmission electron microscope (TEM, operated at 200 kV). An edge of the HB sheet is presented in Figure 3d, providing concrete evidence of the formation of he 2D sheets of borophane. To further characterize the flakes of borophane, we used scanning electron microscopy (SEM) and an electron probe microanalysis (EPMA). Flakes with sizes of 10 µm are shown in the right panel of Figure 3e. Moreover, the elemental mapping in Figure 3e confirms the absence of possible impurities and byproducts, including boric acid. The HB flakes are nonuniform with various thicknesses.

**Figure 4:**
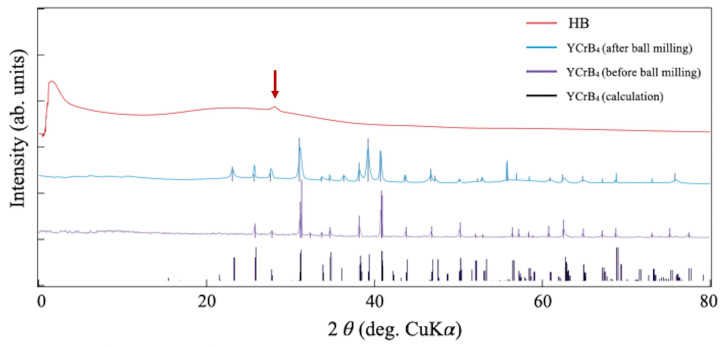
A collection of XRD patterns of the ${\mathrm{YCrB}}_{4}$ crystals (before and after ball milling) and the HB powder. An arrow indicates a diffraction peak of the HB sample.

Figure 4 shows a collection of X-ray diffraction (XRD) patterns of the HB and ${\mathrm{YCrB}}_{4}$ samples. Diffraction peaks of the ${\mathrm{YCrB}}_{4}$ crystals were found at angles, as expected. One can also capture the differences in the XRD signals of ${\mathrm{YCrB}}_{4}$ before and after ball milling. After the process, the peak width became broader and new peaks appeared. These additional features indicate generations of the small crystals with more random orientations. This confirms that the ball milling procedure successfully grinds down crystals of the mother material and reduces their sizes for efficient ion exchange reaction. An XRD pattern of the HB sample, in contrast, shows broad features with a minor diffraction peak at 2θ = 28.1${}^{\circ }$ (±0.2${}^{\circ }$) (an arrow in the figure). Based on Bragg’s condition, the spacing corresponds to 3.17 Å (±0.03 Å). These results suggest that the HB sheets do not feature a long-range order but partially stack with each other with the interlayer spacing of 3.17 Å (±0.03 Å). This is consistent with the microscopic images of the powder samples that show the flexible nature of the sheet. It is, thus, reasonable to consider that the HB material essentially has an amorphous phase with partial stacking of the layers in contrast to a crystalline phase of ${\mathrm{YCrB}}_{4}$. This is likely due to the peeling-off of boron layers by hydrogen adsorption during the reaction, which destroys the long-range order, rather than replacing metal and hydrogen atoms in the crystal framework.

### 2.2. Enhancement of the HB Product by the Acid

**Figure 5:**
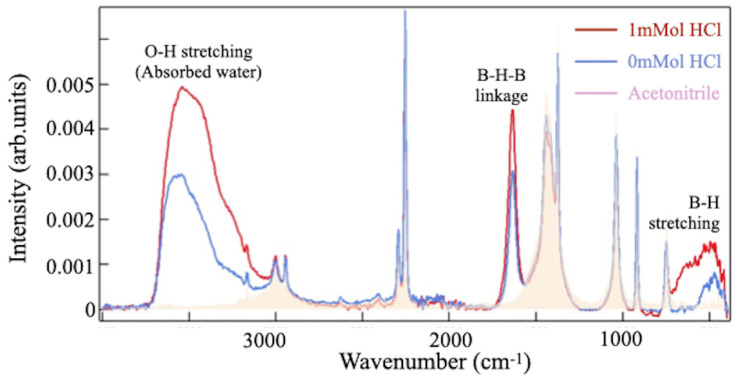
Comparison of the FT-IRAS spectra from the sample filtrate synthesized with (red curve) and without (blue curve) 1 mL of hydrochloric acid. For reference, the spectrum of the acetonitrile solvent (yellow shadow) is displayed.

Figure 5 compares the FT-IRAS spectra over a range of 450–4000 cm^−1^, for the solvent (acetonitrile) and two types of reaction solutions, with and without hydrochloric acid, after one hour. The spectral peaks of the sample at 2250, 1450, 1050, 900, and 750 cm^−1^ match those of the solvent and are therefore assigned to the vibration modes of acetonitrile. The prominent peaks at around 640, 1640, and 3000–3500 cm^−1^ originate from the reaction products and were enhanced when the solution with hydrochloric acid was added. Two peaks at 640 and 1640 cm^−1^ are attributed to the HB samples [16,20,22,23,24]. The broad peak at 3000–3500 cm^−1^ is ascribed to the O-H stretching mode of the water molecule in the hydrochloric acid that remained in the sample. It is noteworthy that an IRAS peak around 1600 cm^−1^ may be attributed to vibrations of the H-O-H bending mode [25]. However, the peak intensity appears differently in previous reports, where the peak of the H-O-H bending mode appears much smaller in comparison to that of the O-H stretching mode [25]. Therefore, one can assign the peak at 1600 cm^−1^ as the B-H-B linkage mode [16,20,22,23,24]. In addition, the peak at 640 cm^−1^ is close to both the B-H stretching and B-B skeletal vibrational bonds, which are present in the borides [16,20,22,23,24,26,27]. In the previous reports, the peaks were assigned to the B-H stretching mode [16,20,22,23,24]. In the powder sample, the IRAS signal at 640 cm^−1^ is associated with the other peak at 2500 cm^−1^ [20]. When the HB powder is put back into the acetonitrile solution, the peak becomes undetectable. The FT-IRAS signal is ascribed to the stretching mode of the free terminal B-H bonds, located at the edges of the HB sheet. The absence of the signal at 2500 cm^−1^ in the liquid sample indicates that the free B-H bonds at the sheet edges are likely influenced by the solvent molecules (acetonitrile), making no signal detection at 2500 cm^−1^ in Figure 5. In this study, the peak observed at 640 cm^−1^ in the FT-IRAS spectrum is, thus, attributed to the B-B skeletal vibrational mode of the HB sheet, as previously reported in the literature, at 740 cm^−1^ in pure boron clusters [26]. The slight shift in the wave number can be attributed to the presence of hydrogen in the HB structure, which has an effect on the vibrational modes of the boron atoms. The IRAS signals provide evidence for the formation of the HB sheet, and the enhancement directly indicates an accelerated ion-exchange reaction by the acid.

**Figure 6:**
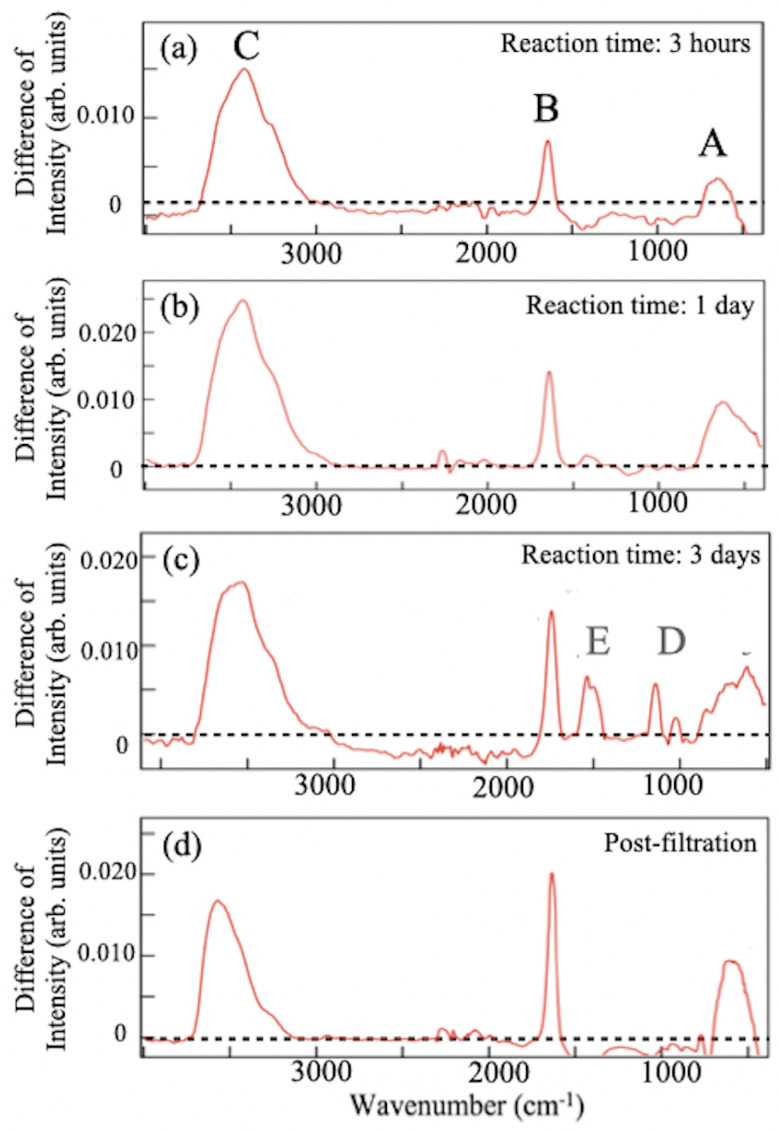
(**a**–**c**) FT-IRAS difference spectra of the solution samples with or without hydrochloric acid (HCl) taken at various reaction times: (**a**) 3 h, (**b**) 1 day, and (**c**) 3 days. (**d**) FT-IRAS difference spectrum of the filtrate after filtration of the sample (**c**). Peaks are labeled with capital letters.

To quantitatively reveal peak enhancements, differences between two FT-IRAS spectra were taken at different reaction times (Figure 6). Specifically, the spectrum of a sample solution without acid (0 mL HCl) was subtracted from that of a sample with 1 mL HCl at different reaction times [see Figure 6a–c], as well as from that of the sample filtrate obtained after three days of reaction [Figure 6d]. The labeled spectral peaks in the figure provide evidence of enhancements in the component due to the addition of HCl to the solution. The peaks labeled as A and B in Figure 6 at approximately 640 cm^−1^ and 1640 cm^−1^ are attributed to the vibrations of the B-B skeletal and B-H-B linkages modes in the HB sheet, respectively [16,22,23,24,26]. The peaks labeled C at ∼3500 cm^−1^ are assigned to the O-H stretching mode of water molecules. The observed peaks confirm the accelerated reaction by hydrochloric acid. In the spectrum of the solution sample after three days of reaction (Figure 6c), one finds the broadening of peak A and the appearance of peaks D at 1050 cm^−1^ and E at 1540 cm^−1^. After filtration, the spectrum shows an absence of both D and E peaks, in addition to the sharpening of peak C. Because the post-filtration process removes boric acid, $B{\left(\mathrm{OH}\right)}_{3}$, from the sample, the spectral changes correspond to the vanishing of the FT-IRAS signal of this impurity. We also note that peaks A and B, which correspond to the HB sheet, become sharper and larger after filtration, suggesting that boric acid, a byproduct of the ion-exchange reaction, affects the neighboring HB sheets. Additionally, the intensity of the O-H stretching bond at 3500 cm^−1^ decreases after filtration, as shown in Figure 6c,d, while the intensity of the 1640 cm^−1^ signal increases, providing further evidence for the assignment of the signal at 1640 cm^−1^ to the B-H-B linkage mode rather than the H-O-H bending mode. The assignments are summarized in Table 1.

**Figure 7:**
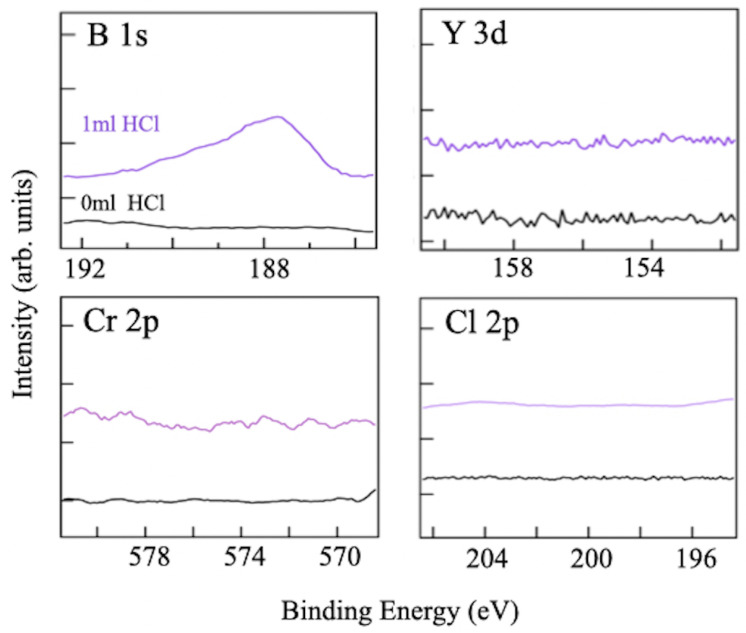
Collection of XPS spectra (purple curves) at the binding energy regions of B 1s, Y 3d, Cr 2p, and Cl 2p core levels for the filtrate sample after three days of reaction with hydrochloric acid (1 mL HCl). For comparison, the individual figures contain the XPS spectra (black curves) of the sample, prepared using the conventional method without hydrochloric acid (0 mL HCl).

To examine the chemical composition of the filtrate sample shown in Figure 6d, XPS measurements were performed on the core-level binding energies of the key elements, B, Y, Cr, and Cl. Figure 7 displays a set of XPS spectra, including a reference spectrum of the filtrate sample prepared via the ion-exchange reaction without hydrochloric acid. After three days of reaction, no detectable amount of any compound was produced using the conventional method. In contrast, the new synthesis procedure with hydrochloric acid results in the appearance of a B 1s peak at a binding energy of 187.6 eV, providing evidence for the formation of sheets of hydrogen boride [16,18,20,22]. The XPS chemical shift of negatively charged boron, in combination with the detected HB bonds in FT-IRAS, clearly indicates the formation of the HB sheet. Additionally, the TEM image of the sample in Figure 3 further supports that it is not composed of elemental boron, as elemental boron sheets cannot exist in the air due to oxidation. Moreover, no signal of the Y 3d and Cr 2p core levels means that there is no remnant of the ${\mathrm{YCrB}}_{4}$ crystal, Y cation, or Cr cation in the sample. In addition, the absence of the Cl 2p peak indicates that the counter ion in hydrochloric acid does not make any compound as a byproduct.

**Figure 8:**
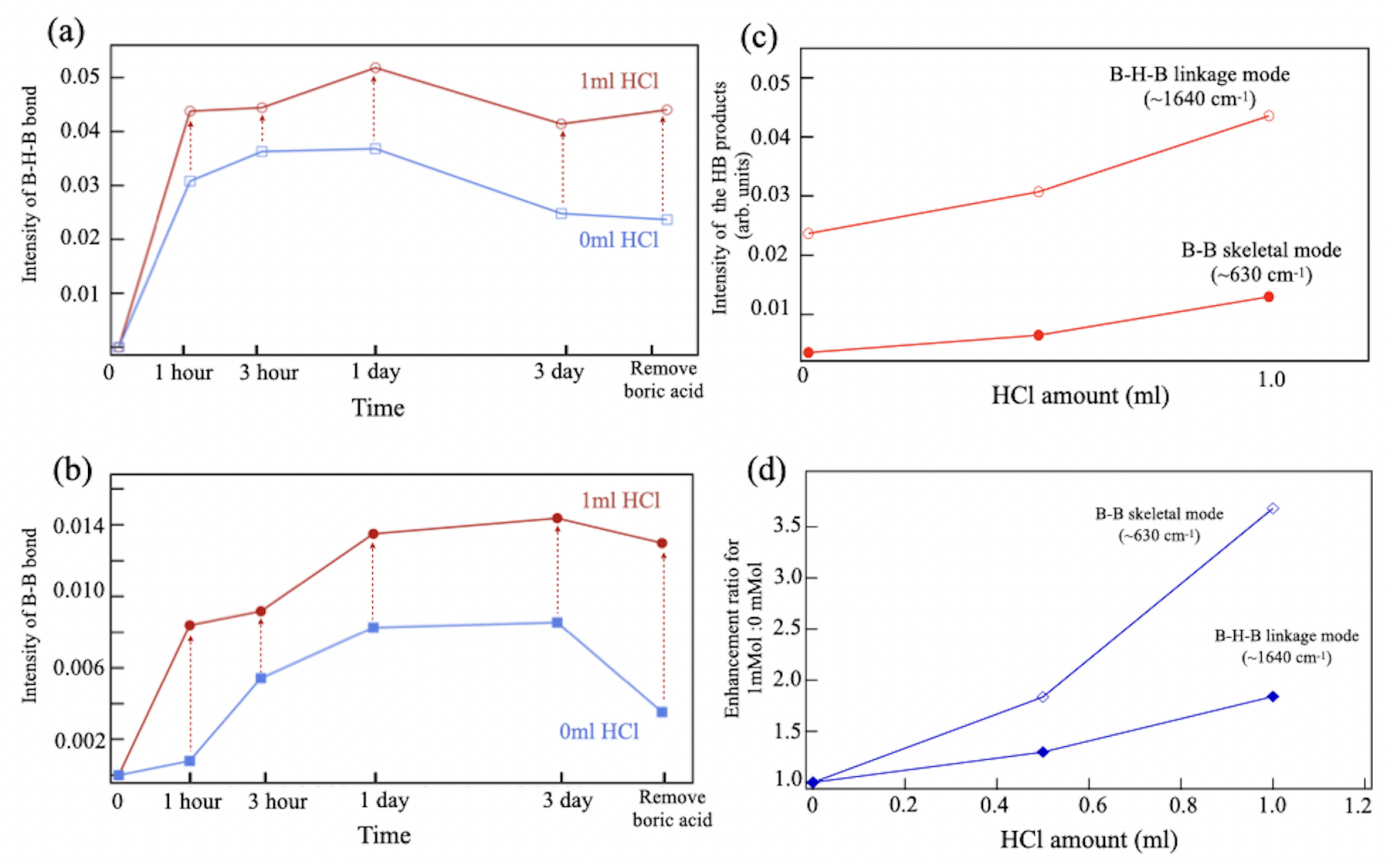
(**a**,**b**) Variations of the FT-IRAS intensity at around (**a**) 630 cm^−1^ (B-B skeletal mode) and (**b**) 1640 cm^−1^ (B-H-B linkage mode) at steps in the synthesis for different reaction times and post-treatments. (**c**,**d**) Changes in (**c**) intensity and (**d**) the ratio of the FT-IRAS signals around 630 and 1640 cm^−1^ with hydrochloric acid concentrations.

Figure 8a,b show the variations in the intensity of the FT-IRAS peaks around 1640 cm^−1^ (B-H-B linkage mode) and 630 cm^−1^ (B-B skeletal mode) for the ion-exchange reaction processes with and without hydrochloric acid. The addition of acid clearly enhances the intensity of both peaks. Moreover, the modes exhibit contrasting behavior. The signal from the B-B skeletal mode increases consistently throughout the process, while the B-H-B linkage mode saturates at an early stage and decreases after several days of reaction. Given that boric acid was identified at this stage in synthesis [Figure 6c], the impurity likely intervenes in the formation of the HB sheet. The product has also shown its increase with the amount of hydrochloric acid (Figure 8c). The enhancement can be quantified by taking ratios of the FT-IRAS intensity at the vibration modes from the data obtained with and without HCl. Increments in the ratio [Figure 8d] are larger for the B-B skeletal mode than for the B-H-B linkage mode. The result indicates that during hydrogenation in the ion-exchange reaction, the formation of the B-H-B bond on the HB sheet begins with B-B bonding, and molecules of boric acid most likely intervene in the subsequent generation of the three-center two-electron (3c-2e) scheme in the B-H-B bond.

With the addition of HCl, the production yield of the reaction significantly improved from around 20% (0 mL HCl), 34% (0.5 mL HCl) to over 50% (1 mL HCl). These results provide evidence for the accelerated synthesis of borophane with 5- and 7-membered boron rings through the use of hydrochloric acid. The use of the acid in HB synthesis has been most notably conducted for the honeycomb borophane with 6-membered boron rings. The synthesis was made through the ion-exchange reaction of ${\mathrm{MgB}}_{2}$ and is accelerated by formic acid [22]. In the present research, the synthesis of 5,7-HB was examined with formic acid but the reaction process and production yield remained unchanged. The difference arises because the mother materials of metal borides react differently with the acid type. The right combination is the key factor in synthesizing the variety of HB sheets through the acid-accelerated ion-exchange reaction.

### 2.3. Analysis of the Precipitate Residue after the Reaction

**Figure 9:**
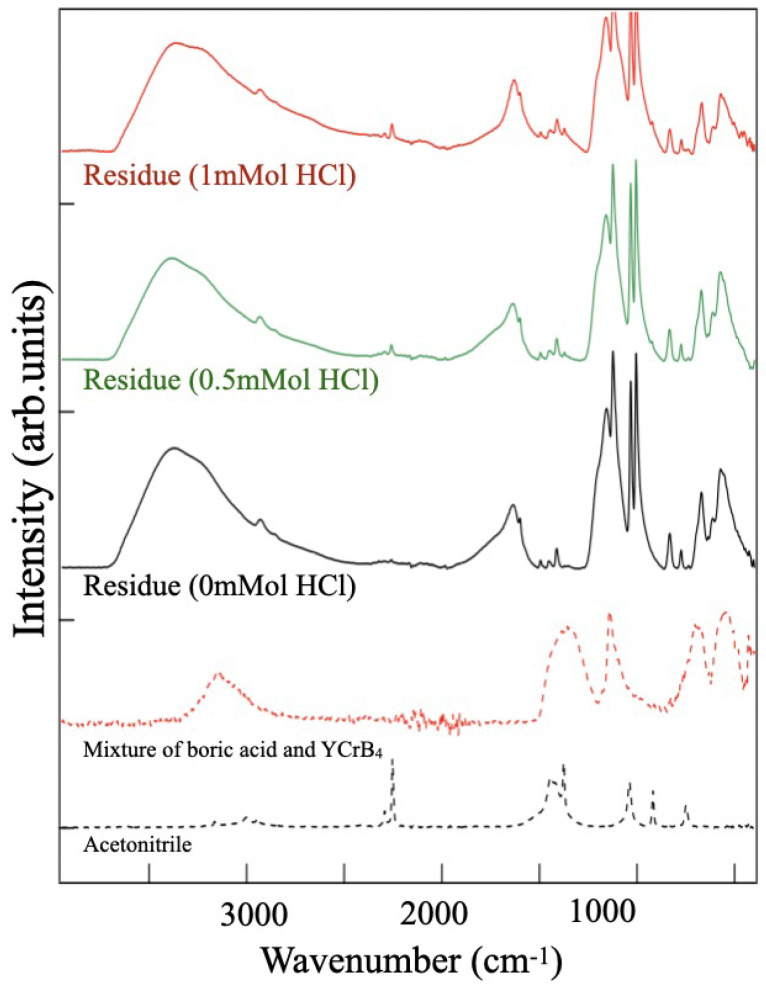
FT-IRAS spectra of the residue obtained from different amounts of HCl, taken after three days of reaction. Reference spectra of a mixture (boric acid and YCrB4) and the acetonitrile solvent are also shown.

The addition of hydrochloric acid in the ion-exchange reaction results in an apparent separation of the solution and the precipitate [Figure 2b]. Drying the precipitate results in a solid residue, Figure 2c, which allows us to handle it for analysis or post-treatment. Figure 9 shows a collection of the FT-IRAS spectra of the various types of the residue obtained after three days of reaction. For comparison, the figure provides the spectra of the solvent (acetonitrile). All residues have similar spectral features, share the same origins, and contain no signal of the volatile solvent. Peaks are found at 630 and 1640 cm^−1^, which can be assigned to the vibration modes of the HB sheet (Table 1, peaks A and D). The spectral appearance implies the existence of 5,7-HB in the residue. The other vibration peaks at 1000–1500 cm^−1^ (D) and the broadband at 3500 cm^−1^ (E) indicates that there are also compounds with O-B and O-H bonds, in comparison with the references. In Figure 9, a spectrum of a mixture of boric acid and ${\mathrm{YCrB}}_{4}$ is given as a reference to make comparisons with the residue spectra. The main features in the spectra of the residue show similarities with those of the starting materials, ${\mathrm{YCrB}}_{4}$, and the byproducts, such as boric acid, $B{\left(\mathrm{OH}\right)}_{3}$, confirming their presence in the residue.

**Figure 10:**
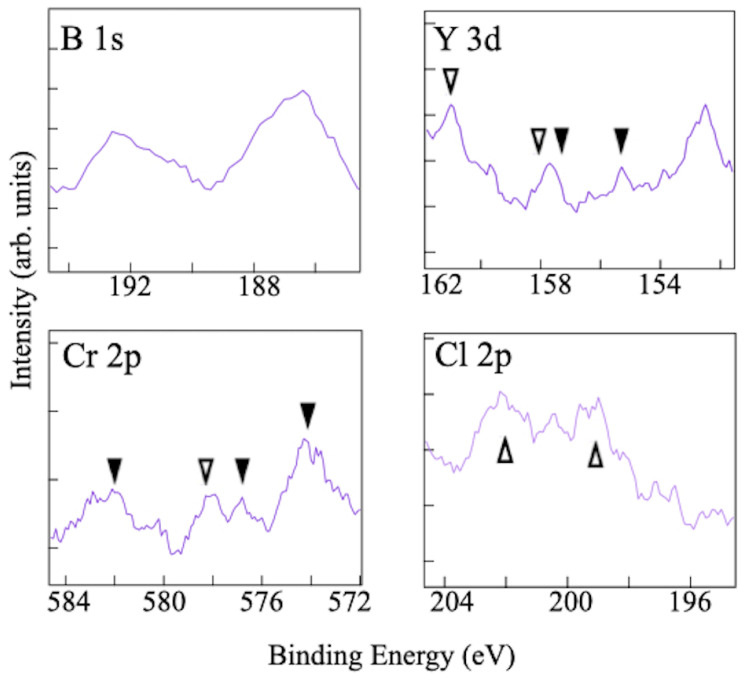
XPS spectra of the residue, taken after three days of reactions, for the core levels of B 1s, Y 3d, Cr 2p, and Cl 2p. Peaks of ${\mathrm{YCl}}_{3}$ and ${\mathrm{CrCl}}_{3}$ are labeled by open triangles [30,31], whereas remnants of ${\mathrm{YCrB}}_{4}$ are labeled with black triangles [16,18,20].

After confirming the absence of Y, Cr, and Cl atoms in the filtrate sample of the HB sheet, we conducted an XPS analysis of the residue (Figure 10) to trace the elements. The appearance of the Cl core-level peaks indicates the production of chlorides and, accordingly, peaks of ${\mathrm{YCl}}_{3}$ and ${\mathrm{CrCl}}_{3}$ are found together with the remnant ${\mathrm{YCrB}}_{4}$ in the individual spectra [30,31]. Two peaks are observed in the B 1s core-level spectrum at 187 and 192 eV. The former corresponds to negatively charged boron atoms that can be assigned to HB and ${\mathrm{YCrB}}_{4}$, whereas the latter is assigned to positively charged boron of boric acid. These results indicate that Y and Cr atoms are efficiently captured as chlorides or are kept in the mother material that remains. By gathering the residue after the reaction, the Y and Cr atoms can be collected easily in solids. Then they become raw materials to produce ${\mathrm{YCrB}}_{4}$, which can be used in the next synthesis of the 5,7-HB. Thus, this HCl-assisted ion-exchange reaction has the advantage of recycling rare-earth metal.

**Figure 11:**
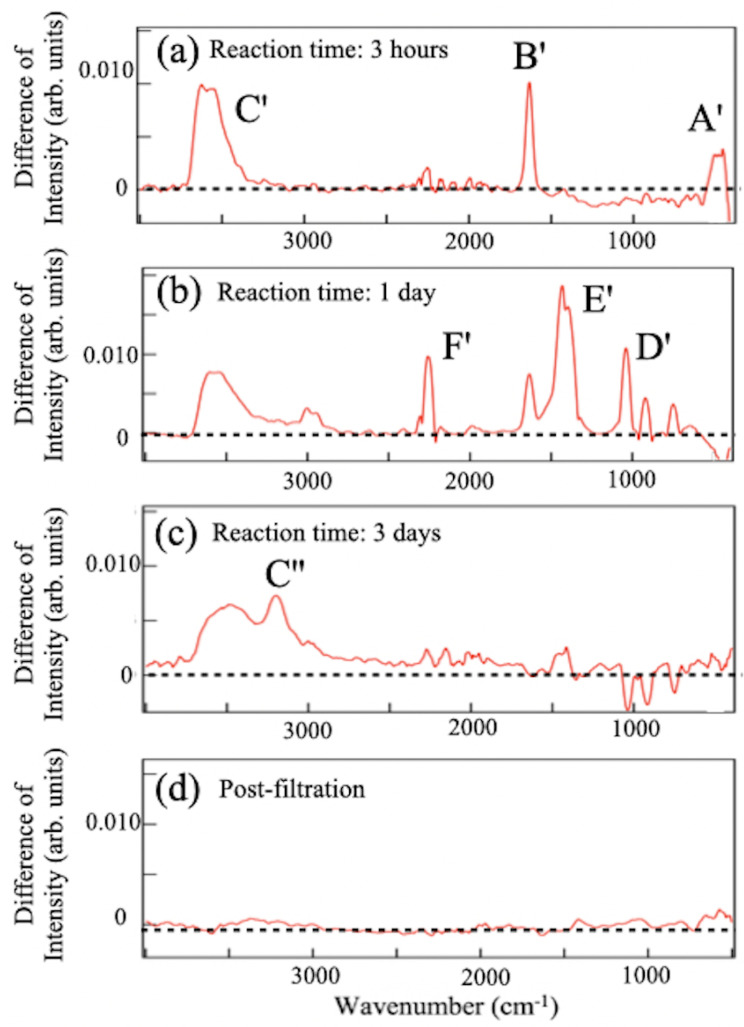
Difference spectrum of FT-IRAS, obtained by subtracting the spectrum of the normal ion-exchange sample from the spectrum of the acid-ion exchange sample, synthesized from bulk ${\mathrm{YCrB}}_{4}$. The measurements were taken at (**a**) 3 h, (**b**) 1 day, (**c**) 3 days, and (**d**) post-filtration. Peaks of the vibrational modes are labeled.

To deepen the understanding of the ion-exchange reaction, the synthesis was made without the ball-milling process, leaving the mother crystals of ${\mathrm{YCrB}}_{4}$ at mm size. Figure 11 shows several FT-IRAS difference spectra taken at separate reaction times and after the post-filtration of a sample after three days of reaction. The assignments of FT-IRAS peaks are summarized in Table 2. Peaks corresponding to the B-B skeletal (A’), as well as B-H-B linkage (B’) vibrational modes of the HB sheet were observed after a three-hour duration at 639 and 1627 cm^−1^, respectively. Around 3500 cm^−1^, a peak appears that is associated with the O-H stretching mode, C’. The spectral appearances are similar to those in Figure 6a,b. However, after one day, the spectrum is governed by peaks of boric acid, D’ and E’, at 1050 and 1512 cm^−1^, respectively, with the weak B’ peak. The FT-IRAS spectra show the presence of the feature at 2500 cm^−1^ that can be assigned to the free B-H bonding mode [20]. Since the spectral appearance is associated with the reduction of the A’ and B’ signals, it may be ascribed to broken fragments of the small HB molecules from the sheet. Eventually, after three days, no appearance of the FT-IRAS signal of the boron material was detected in the solution or the filtrate. The products precipitate at the bottom of the reaction beaker.

These results reveal significant details of the ion-exchange reaction underlying the synthesis of 5,7-HB. From Figure 6 and Figure 11, one finds that the production of the HB sheet is followed by that of boric acid and the crossover time depends on the size of the mother material, ${\mathrm{YCrB}}_{4}$. The reaction time required for HB synthesis is longer when the crystal size is reduced through ball milling. This is because as the crystal size decreases, the ratio between surface area and volume increases, and the ion-exchange reaction primarily occurs at the surface of the crystal. Exfoliation results in a larger amount of product when the crystal size is smaller. After the reaction, molecules of borophane and boric acid precipitate and mix to form the residue. Therefore, to obtain high-purity products of 5,7-HB, the reaction time needs to be optimized and depends on the size of the ${\mathrm{YCrB}}_{4}$ crystal and the desired level of filtrate extraction.

## 3. Materials and Methods

### 3.1. Synthesis Methods

Polycrystalline ${\mathrm{YCrB}}_{4}$ was fabricated by the arc melting method [20]. Rare earth metals, Y (890 mg), Cr (520 mg), and B (432 mg) were loaded in a Cu hearth with an atomic ratio of 1:1:4, and melted under the Ar atmosphere. In total, the resulting polycrystals were 1.632 g and were milled in an attritor by 5 mm diameter ${\mathrm{ZrO}}_{2}$-based balls, reduced to µm-sized powders. The crystalline features were confirmed by X-ray diffraction (XRD).

The ion exchange reaction was prepared as follows. 100 mg powders of ${\mathrm{YCrB}}_{4}$ were mixed with a 20 mL cation ion-exchange resin (0.5–1.0 mm, Amberlite IR120B hydrogen form, Organo Corp., Tokyo, Japan) into a 50 mL solvent of acetonitrile (99.5%, Wako Pure Chemical Industries, Ltd., Osaka, Japan). Then, 1 mol/L HCl (0 mL/0.5 mL/1 mL) of hydrochloric acid was added to the solvent. The reaction was continuously stirred (250 rpm) using a magnetic stirrer under inert gas (Ar) at room temperature. The filtration was conducted after ion exchange reaction (0.2 µm pore filter, Omnipore Membrane Filters, Merck Millipore, Billerica, MA, USA).

### 3.2. Characterization Methods

XRD (SmartLab 3 kW) measurements were conducted with CuKα operated at 40 kV and 30 mA at room temperature in an ambient atmosphere. The TEM (JEOL-2100) was operated with 200 keV. The SEM-EPMA (JEOL JXA-8530F) was conducted with 20 keV.

FT-IRAS measurements were performed to investigate the vibrational modes of the sample that evidence the formation of vibrational bonds in the HB sheet. The experiment was conducted using a commercial system (BRUKER ALPHA II FT-IR spectrometer). The FT-IRAS spectra were recorded by applying the attenuated total reflectance method and using a prism holder that was filled with a solution of either a sample of the filtrate or a dried sample of the residue. All measurements were made at room temperature.

Elemental compositions and chemical states of the samples were examined by XPS. Measurements were performed with the incident X-ray beam of energy hν = 1253.6 eV (Mg Kα) and a photoelectron analyzer (Scienta Omicron DA30-L-8000). Before their transfer into the UHV chamber, samples were annealed at 373 K in nitrogen gas to remove water molecules as they violate the UHV condition for XPS measurements. The XPS spectra were recorded at room temperature.

## 4. Conclusions

We discovered that adding hydrochloric acid to the ion-exchange reaction significantly improves the efficiency of producing topological borophane, a novel 2D material. The production yield increases to 50% compared to the reaction without the acid (20%). The high-purity borophane sheets are dispersed in solution and can be extracted through filtration. The metal atoms of the mother material, ${\mathrm{YCrB}}_{4}$, Y and Cr, form metal chlorides and precipitate as a residue along with byproducts such as boric acid and remnants of ${\mathrm{YCrB}}_{4}$. The use of hydrochloric acid provides advantages in both accelerating production yield and isolating high-purity products. The mass-production method developed in this research paves the way for expanding fundamental research on novel topological 2D materials that may have a wide range of industrial applications.

## Figures and Tables

**Table 1:** Summary of the assignment of the FT-IRAS peaks (Figure 6); the assignments are based on references [16,22,23,24,26,27] (unit: cm^−1^).

Label	3 h	1 day	3 days	Post-Filtration	Reference (Vibration Mode)
A	634	631	635	632	B-B skeletal [26,27]
B	1652	1654	1645	1639	B-H-B linkage [16]
C	3232	3524	3014	3429	O-H stretching [28]
D	-	-	1050	-	H-O-B deforming [28]
E	-	-	1540	-	B-O stretching [29]

**Table 2:** Summary of the assignments for the FT-IR peaks (Figure 11, sample synthesized from bulk ${\mathrm{YCrB}}_{4}$); the assignments are based on references [16,22,23,24] (unit: cm^−1^).

Label	3 h	1 day	3 days	Post-Filtration	Reference (Vibration Mode)
A’	639	-	-	-	B-B skeletal [26]
B’	1627	1642	-	-	B-H-B linkage [16]
C’	3540	3550	3500	-	O-H stretching [29]
D’	-	1050	-	-	B-H stretching [22,29]
E’	-	1512	-	-	B-O stretching [28]
F’	-	2364	-	-	B-H stretching [23]
C”	-	-	3225	-	O-H stretching [29]

## Data Availability

Not applicable.
